# Phenotypic and Genetic Comparison of a Plant-Internalized and an Animal-Isolated *Salmonella* Choleraesuis Strain

**DOI:** 10.3390/microorganisms9081554

**Published:** 2021-07-21

**Authors:** Irene Esteban-Cuesta, Mirian Labrador, Katharina Hunt, Sven Reese, Jennie Fischer, Karin Schwaiger, Manfred Gareis

**Affiliations:** 1 Chair of Food Safety, Veterinary Faculty, LMU Munich, 85764 Oberschleissheim, Germany; huntkatharina@gmail.com (K.H.); karin.schwaiger@vetmeduni.ac.at (K.S.); manfred.gareis@posteo.de (M.G.); 2 Departamento de Producción Animal y Ciencia de los Alimentos, Veterinary Faculty, Instituto Agroalimentario de Aragon-IA2, University of Zaragoza-CITA, 50013 Zaragoza, Spain; mlabrad@unizar.es; 3 Chair of Anatomy, Histology and Embryology, Department of Veterinary Sciences, Faculty of Veterinary Medicine, LMU Munich, 80539 Munich, Germany; s.reese@anat.vetmed.uni-muenchen.de; 4 National Salmonella Reference Laboratory, Unit Food Microbiology, Host-Pathogen-Interactions, Department of Biological Safety, German Federal Institute for Risk Assessment (Bundesinstitut für Risikobewertung—BfR), 12277 Berlin, Germany; jennie.fischer@bfr.bund.de; 5 Unit of Food Hygiene and Technology, Institute of Food Safety, Food Technology and Veterinary Public Health, University of Veterinary Medicine Vienna, 1220 Vienna, Austria

**Keywords:** *Salmonella*, adaptation, endogenous, fresh produce, vegetables, foodborne pathogen, food safety

## Abstract

Contamination of fresh produce with human pathogens poses an important risk for consumers, especially after raw consumption. Moreover, if microorganisms are internalized, no removal by means of further hygienic measures would be possible. Human pathogenic bacteria identified in these food items are mostly of human or animal origin and an adaptation to this new niche and particularly for internalization would be presumed. This study compares a plant-internalized and an animal-borne *Salmonella enterica* subsp. *enterica* serovar Choleraesuis aiming at the identification of adaptation of the plant-internalized strain to its original environment. For this purpose, a phenotypical characterization by means of growth curves under conditions resembling the indigenous environment from the plant-internalized strain and further analyses using Pulsed-field gel electrophoresis and Matrix-assisted laser desorption ionization time of flight spectrometry were assessed. Furthermore, comparative genomic analyses by means of single nucleotide polymorphisms and identification of present/absent genes were performed. Although some phenotypical and genetic differences could be found, no signs of a specific adaptation for colonization and internalization in plants could be clearly identified. This could suggest that any *Salmonella* strain could directly settle in this niche without any evolutionary process being necessary. Further comparative analysis including internalized strains would be necessary to assess this question. However, these kinds of strains are not easily available.

## 1. Introduction

In recent years, fresh fruit and vegetables for raw consumption have increasingly been related to foodborne disease outbreaks. Contamination of these products with pathogenic microorganisms poses a special risk, as no hygienic measures are applied in order to inactivate the pathogens [1,2]. Many studies have addressed the contamination of crops with human pathogenic Enterobacteria and plants have become a normal step in the lifecycle of these microorganisms [3,4,5,6]. In addition to the surface contamination of these food items, internalization of human pathogenic bacteria in plants and their fruits has also been addressed [7,8,9,10,11,12]. Internalization seems to depend on both the microorganism and the plant variety, with the immune system of each plant able to more effectively recognize certain strains than others, hence triggering a stronger or weaker immune response [8,9,13]. This would facilitate the colonization of the inner parts of plants by certain microorganisms.

Many of the opportunistic and pathogenic microorganisms related to plants that are important in food safety are of animal and human origin. Therefore, these microorganisms might have somehow adapted to the new conditions of the plant environment and both structural and genetic modifications might have been needed for internalization [13,14]. These hypothetical modifications might then allow strains adapted to the plant environment to survive, grow better or even internalize under the correspondent conditions than other strains of animal origin and without previous contact to the plant environment, which would not be adapted to plants and therefore be unable to colonize them.

*Salmonella* spp. are important zoonotic and foodborne pathogens, which belong to the *Enterobacteriaceae* family and mainly cause gastrointestinal diseases in humans. The EU Member States reported 5175 foodborne outbreaks in 2019, in which *Salmonella* spp. was the most detected. Especially strains of *Salmonella enterica* subsp. *enterica* (*S.*) cause important economic and public health problems [15]. In a previous study [12], *S.* Choleraesuis was isolated from the pulp of a sterile opened muskmelon. This strain was plant-internalized and therefore already able to colonize edible parts of the fruit and could therefore hypothetically be plant-adapted. Therefore, this strain poses an opportunity to clarify whether certain differences between plant-adapted and not plant-adapted strains are present.

In order to evaluate these possible phenotypical differences, two isolates of *S.* Choleraesuis were analyzed. The plant-internalized strain from the pulp of a Galia melon and a second *Salmonella* strain of the same serovar, but isolated from wild boar, which was used as a strain of animal origin.

Characterization of both strains was performed by assessing classic bacterial growth curves under conditions resembling the original environment of the plant-internalized strain, muskmelons. Different broths, temperatures and pH conditions, addition of malic and/or citric acid and growth in the presence of different sugars and combinations of these were used for this purpose. Additional analyses were performed by means of pulsed-field gel-electrophoresis (PFGE) and Matrix-assisted laser desorption ionization–time of flight mass spectrometry (MALDI-TOF MS). Finally, a pangenome analysis allowed a comparison of the presence/absence of genes, which gave a genetic insight together with a single nucleotide polymorphism (SNPs) study.

## 2. Materials and Methods

### 2.1. Selected Bacterial Isolates 

In this study, two strains of the serotype *Samonella enterica* subsp. *enterica* serovar Choleraesuis were analyzed, being one isolate of plant origin isolated from the pulp of muskmelons (var. Galia; [12]), and one of animal origin from wild boar (septicemic infection of inner organs), provided by the German National Reference Laboratory for *Salmonella*.

### 2.2. Bacterial Growth Curve

In order to analyze possible adaptations of the plant-internalized strain to the plant environment, the growth potential of both strains was analyzed under specific established conditions and classical bacterial growth curves were recorded. These conditions were intended to represent those of muskmelons, as this was the original environment of the strain of plant origin. These analyses were performed in three technical replicates. The resulting pH was 7.14 ± 0.04 for all solutions, excluding the experiments with adjusted pH (see below).

#### 2.2.1. Bacterial Solution

For each assay, one loop (10 µL) of each strain was taken from blood agar (OXOID, Wesel, Germany) and inoculated in a Brain Heart Infusion broth (BHI; VWR Chemicals, Leuven, Belgium) and incubated at 37 °C. After approximately 24 h, the bacterial concentration reached approximately 9.0 log_10_ CFU/mL (preliminary data). The BHI suspensions with the analyzed *Salmonella*-strains were serial diluted, so that after inoculation in 100 mL test solution, a final concentration of approximately 1.0 log_10_ CFU/mL was reached and set as the initial point (t_0_) for the growth curve.

#### 2.2.2. Mashed Melon, Tryptic Soy Broth and Buffered Peptone Water

Growth in mashed melon (MM), Tryptic Soy Broth (TSB; Merck KGaA, Darmstadt, Germany) and buffered Peptone water (BPW; Merck KGaA, Germany) was assessed. Mashed melons were prepared using Galia melons. Fruits were first opened under sterile conditions, as described in Esteban-Cuesta [12], and then blended. Absence of *Salmonella* spp. in the purchased melons was confirmed according to the ISO Norm 6579-1:2017. Incubation during analysis took place for each broth and strain at 5 °C (refrigeration temperature), 22 °C (ambient temperature), and 37 °C (optimal growth temperature for *Salmonella* spp.).

#### 2.2.3. Variation in pH Conditions

BPW (100 mL) was settled at pH-values from 3.5 to 8.5 in 0.5 steps by means of Sodium hydroxide solution (NaOH; Merck KGaA, Darmstadt, Germany) and Hydrochloric acid (HCl; Merck KGaA, Darmstadt, Germany). Incubation of the inoculated media took place at 37 °C for growth curves assessment.

#### 2.2.4. Addition of Sucrose, Fructose and Glucose

Growth curves were also assessed in presence of different sugars and the combination of these. Sucrose (S; 60.0 g/L), fructose (F; 20.0 g/L), glucose (G; 11.0 g/L) and a combination of all three sugars in the same amount (Mix) were dissolved in 100 mL BPW and incubated at 37 °C for the growth curves. Sugar concentrations were established according to previous analyses of the sugar content of muskmelons [16].

#### 2.2.5. Addition of Malic and Citric Acid

Both malic (MA) and citric acid (CA) were mixed in 100 mL BPW at two different concentrations: one in an approximately natural concentration present in the pulp of muskmelons (MA: 2.0 ppm, CA: 3.5 ppm, according to values analyzed by Flores [16]) and one with fivefold higher of this concentration (MA: 10 ppm, CA: 17.5 ppm). Incubation of the inoculated media was performed at 37 °C.

#### 2.2.6. Sampling

Samples from inoculated testing media were taken for the conditions mentioned at the following time intervals from the time of inoculation of the culture: directly after inoculation (t_0_), after 6 h (t_6_), after 9 h (t_9_), after 12 h (t_12_), after 24 h (t_24_), after 48 h (t_48_) and after 72 h (t_72_). The periodic samplings were plated on brilliant-green phenol-red lactose sucrose (BPLS) agar (Merck KGaA, Darmstadt, Germany) and incubated for 24 h at 37 °C to determine viable counts (as colony-forming units per ml; CFU/mL).

#### 2.2.7. Statistical Analysis

Statistical analyses for identifying significant associations for the tested growth conditions between both strains (plant origin and animal origin), were carried out using SPSS, version 26 (IBM Deutschland GmbH, Munich, Germany). A generalized linear model (GLM) was used for analysis. To consider the multiple comparisons, the Bonferroni correction was applied. *p* < 0.05 was considered statistically significant. 

### 2.3. MALDI-TOF MS

An Autoflex Speed MALDI-TOF/TOF MS (Bruker Daltonics GmbH, Bremen, Germany) was used for measurement, which was performed in the linear positive mode (*m*/*z* 2–20 kDa) with the Bruker Daltonics GmbH AutoX method (MBT_AutoX) using the software flexControl v.3.4.135 for monitoring and “MBT (MALDI Biotyper) Compass v4.1.80” for identification of the microorganisms. The following parameters were set: basic laser settings: laser energy 30–40% (global attenuator offset 22%) with a matrix blaster of 40 laser shots with 60% laser energy; high voltage settings: ion source 1: 19.5 kV; ion source 2: 18.4 kV; lens: 8.0 kV; pulsed ion extraction set to 380 ns. The Bacterial Test Standard (BTS) from Bruker Daltonics GmbH & Co. KG (Bremen, Germany; mass range: 3637.8–16,952.3 Da) was used as the calibration standard. Calibration was performed weekly.

Each biological replicate was spotted 8 times and measured three times on different days to guarantee reliable results and exclude daily influences. The obtained mass spectra were evaluated as follows: minimum resolution: 400 arb.u., signal to noise: 2, peak intensity: 600 arb.u., evaluated peaks: 4–10 kDa; accumulation of 1200 satisfactory shots in 200 steps done by random walk and maximum 400 shots in a raster spot. The measurement was aborted if 20 subsequently failed judgments were obtained (null spectrum). 

Mass spectra were visualized and edited using the software flexAnalysis v.3.4.76. The statistical analysis was performed using the “*p* value tta” sort mode of the ClinProTools 3.0 software (Bruker Daltonics GmbH & Co. KG). Only peaks that fulfilled the previous requirements were annotated as mass peaks.

### 2.4. PFGE Typing

PFGE analysis was performed according to the PulseNet standardized laboratory protocol (Pulse-Net, Centers for Disease Control and Prevention, Atlanta, GA, USA) (Ribot et al., 2006) with minor changes. Both isolates were grown on tryptic soy agar at 37 °C and then bacterial cells were suspended into a cell suspension buffer (100 mM Tris, 100 mM EDTA, pH 8.0) to an optical density of 0.8–1.0 at OD610. Proteinase K (20 µL) was added to 400 µL of the suspension and 400 µL of molten (54 °C) 1% Biozym Gold Agarose. After mixing, 90 µL was dispensed into disposable plug molds, five times for each sample. Once solidified, the plugs were placed into 5 mL cell lysis buffer (50 mM Tris, 50 mM EDTA, pH 8.0, 1% Sarcosyl) and 25 µL Proteinase K, followed by 2 h incubation at 54 °C in a shaking water bath. The plugs were washed twice in ultrapure sterile water for 15 min in a 45 °C water bath, then washed four times in Tris-EDTA (TE) buffer (10 mM Tris, 1 mM EDTA, pH 8.0) and stored in TE buffer at 4 °C.

For PFGE, the plugs were cut into 3 mm by 10 mm pieces, then incubated in a prerestriction step (180 µL sterile ultrapure water, 20 µL 10× Cut Smart^®^ Buffer (New England Biolabs GmbH, Frankfurt am Main, Germany) and digested in 176.5 µL sterile water, 20 µL 10× Cut Smart^®^ Buffer, 1 µL bovine serum albumin (20 mg/mL), 2.5 µL XbaI (20 U/µL) for 2 h at 37 °C in a ThermoMixer^®^ comfort (Eppendorf AG, Hamburg, Germany). The slices were run in a 1% agarose gel using a Chef Mapper XA (Bio-Rad, USA) in 0.5× Rotiphorese^®^ Tris-borate-EDTA buffer (Carl Roth GmbH+ Co. KG, Karlsruhe, Germany) at 14 °C for 20 h with a switch time 2.16–36.8 s. and a voltage of 6 V/cm. *Xba*I-digested plugs of *S.* Braenderup (ATCC^®^ BAA664™) were prepared as described above and used as standard and were.

The gel was stained with ethidium bromide and PFGE band patterns were visualized by Gel Doc™ gel imaging system (Bio-Rad Laboratories Inc., Herkules, CA, USA). Analysis and generation of dendrograms were performed using BioNumerics version 7.6 (Applied Maths, Austin, TX, USA). Comparisons of the resulting PFGE band patterns were made using Dice coefficients and the Unweighted Pair Group Method with Arithmetic mean (UPGMA) for clustering, followed by band matching optimization 0.2% and band position tolerance at 0.8%.

### 2.5. Library Preparation and Phylogenetic Analysis

The genomic DNA from both strains was sequenced by means of Next Generation Sequencing (NGS) as described in Esteban-Cuesta [17]. All sequencing parameters of the plant-internalized *S.* Choleraesuis are also available on the same Microbiology Resource Announcement. The sequencing Library of the strain of animal origin was also created with the Nextera DNA Flex Library Prep Kit (Illumina, San Diego, CA, USA) and sequenced with 2× 151 Cycles on a NextSeq Benchtop Sequencer 500 with Mid Output Kit v3 300 cycles (Illumina, San Diego, CA, USA), resulting in 3,180,706 total reads and a 99.6-fold coverage. The generated paired-end reads (Q30 base fraction: 0.87) were trimmed and quality controlled using fastp (v0.19.5; 10), with default parameters (except—length_required 15) and *de novo* assembled using Shovill v1.1.0 [10,11] using SPAdes as assembly method (with options—noreadcorr—depth 100 and otherwise default parameters), using the AQUAMIS Pipeline (https://gitlab.com/bfr_bioinformatics/AQUAMIS, Version 1.2.0; last accessed 26 November 2020); see also Deneke [18]. The assembled *S.* Choleraesuis genome (animal origin) comprised 67 contigs with a total length of 4,733,941 bp and *N_50_* value 177,891.

SNP calling was based on trimmed reads and conducted using the snippySnake pipeline (https://gitlab.com/bfr_bioinformatics/snippySnake, Version 1.0.0, last accessed 26 November 2020); see also Lüth [19] for variant calling (snippy parameters: mapqual 60, basequal 13, mincov 10, minfrac 0, minqual 100, maxsoft 10). NZ_CP007639.1 (*Salmonella enterica* subsp. *enterica* serovar Choleraesuis strain C500 chromosome, complete genome) was used as reference sequences for SNP calling.

In addition, a pangenome analysis was performed using both sequenced libraries to investigate presence/absence of genes. The pangenome was extracted with Roary v3.11.2 (last accessed 17 February 2021) [20] from the previously Prokka-annotated (v1.14.6) GFF3 files [21]. Sequences of the genes identified were again searched on the sequences of the other strain using Geneious Prime^®^ v2020.1.1 to reassure absence in the analyzed genomic DNA of the other strain.

## 3. Results

### 3.1. Bacterial Growth Curves

**Figure 1:**
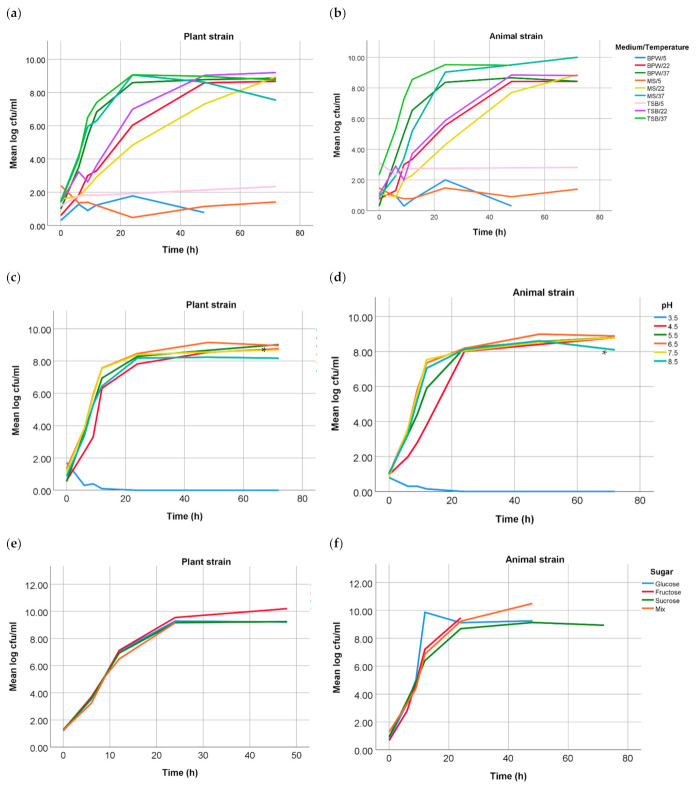

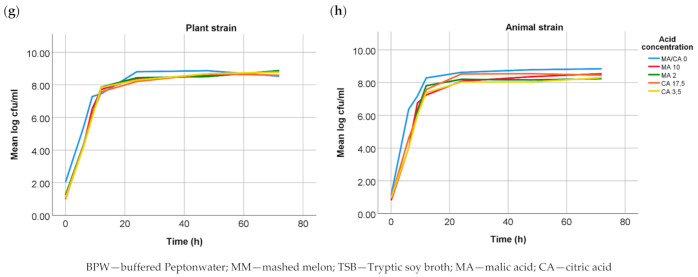
Growth curves of both *Salmonella* Choleraesuis strains under different conditions: growth in BPW, TSB and MM at 5, 22 and 37 °C of the plant (**a**) and the animal (**b**) strain; growth at different pH values in BPW at 37 °C of the plant, where differences at pH 5.5 were significant (*) with a *p*-value of 0.030 (**c**), and the animal strain (**d**); growth in addition of different sugars of the plant (**e**) and the animal (**f**) strain; and growth at different concentrations of malic and citric acid of the plant (**g**) and the animal (**h**) strain.

In the present study, differences between growth curves from both *Salmonella* Choleraesuis strains were performed under different conditions. Growth curves in solutions with the addition of sugars and organic acids showed no significant difference (*p* > 0.05). Additionally, no statistically significant difference was found between the growth curves of both strains in different media and in grounded melon at different temperatures. However, growth analysis at different pH values showed a statistically significant difference (*p* = 0.030) at pH 5.5 (37 °C), the strain of plant origin reached faster the stationary phase than the strain of animal origin. Growth curves for the parameters media and mashed melon at different temperatures, pH-values and organic acids are shown in Figure 1.

### 3.2. MALDI-TOF MS

**Figure 2:**
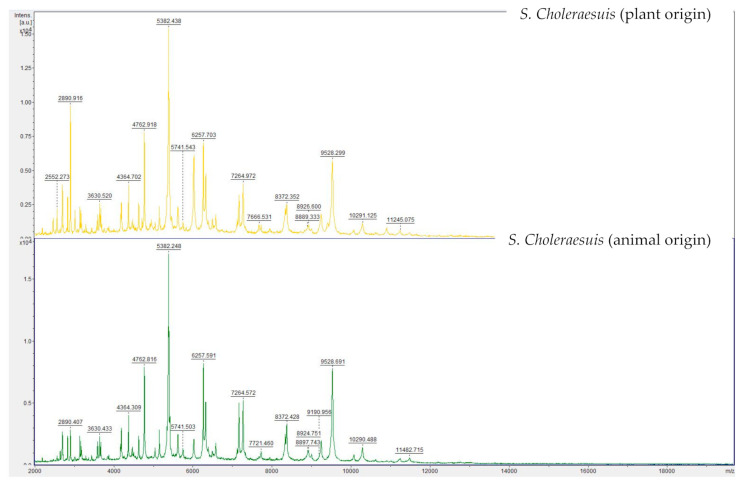
MALDI-TOF MS spectra of both *Salmonella* Choleraesuis strains displayed in flexAnalysis.

Mass spectra were acquired by means of MALDI-TOF MS. Figure 2 shows the spectra of both strains displayed in FlexAnalysis. Differences between both strains were not significant (*p* > 0.05).

### 3.3. PFGE Typing

**Figure 3:**
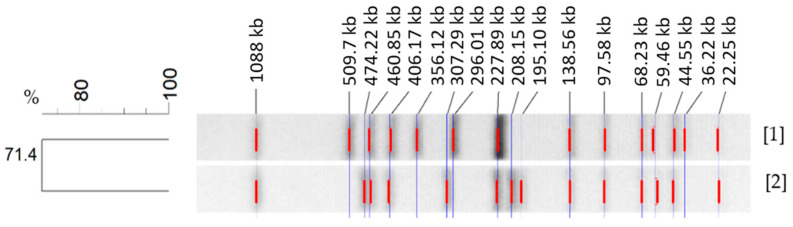
Genetic relatedness of *S.* Choleraesuis strains of plant [1] and animal origin [2] determined by PFGE with restriction enzyme *Xba*I, showing conserved and disparity bands. Cluster analysis of the resulting band pattern was performed using the Dice coefficient and UPGMA with band tolerance 0.8%.

PFGE analysis of both *S.* Choleraesuis strains showed two different *Xba*I-PFGE patterns, which are depicted in Figure 3. These patterns were separated from each other resulting in a 71.4% similarity. This similarity is associated with the fragments conserved within the *Xba*I patterns from each strain. Characterization of both band patterns showed a disparity in a total of eight bands. The conserved bands for both *S.* Choleraesuis were at 1088 kbp, 460 kbp, 406 kbp, 227 kbp, 138 kbp, 97 kbp, 68 kbp, 59 kbp, 44 kbp and 22 kbp.

### 3.4. Comparative Genomics

The SNP-Analysis of both isolates showed an SNP distance of 2081 SNPs from each other while using strain NZ_CP007639.1 as reference.

The pangenome analysis of a total of 4601 genes showed 4465 core genes (i.e., genes shared by 99% of the isolates) and 130 shell genes (i.e., genes in 15% ≤  strains < 95%). From these shell genes, 67 were identified as hypothetical proteins (plant origin, *n* = 26; animal origin, *n* = 41) and 47 were different alleles from a common gene, sharing both strains at least one allele. Finally, both strains differ in 16 genes, which are summarized in Table 1. 

## 4. Discussion

In the present study, we present a phenotypical and genotypic comparison of two *S.* Choleraesuis strains isolated from both plant and animal origin. The aim of these analyses was to examine their differences and evaluate whether an adaptation to the growth in melons could be detected at the phenotypical or genomic level for the strain of plant origin, which was previously plant-internalized.

Growth analysis under the different tested conditions was significantly different for growth at pH 5.5 (*p* = 0.03, 37 °C). While the strain of plant origin reached 8.00 log CFU ml^−1^ after 20 h, the animal strain did not reach it until 22 h. Therefore, the plant strain appeared to grow slightly faster at pH 5.5 than the strain of animal origin. *S. enterica* are facultative intracellular enteropathogens when infecting humans and animals. However, it has been seen that human pathogens usually localize during plant colonization in the intercellular space (apoplast) and do not internalize plant cells [22]. In contrast to the cytoplasmic pH of both plant and animal cells, which would be 7.0–7.5, the overall apoplastic pH in plants is 5.7 [23], which would be similar to the tested condition. Nevertheless, while internalization in animal cells, *Salmonella* spp. resides and replicates inside host cells in membrane-bound compartments called *Salmonella*-containing vacuoles [24]. These can also acidify to between pH 4.0 and 5.0 [25], although this mechanism was shown not to be necessary for the survival of *S.* Typhimurium within macrophages and epithelial cells and not to be activated by all bacteria during infection [25,26,27]. Furthermore, the roots apoplast is in direct contact with the soil [22]. Thus, an adaptation to this pH could facilitate internalization in the plants through this path. 

In the following stage, mass spectra acquired by means of MALDI-TOF MS were also compared. This tool is today regularly used for the identification of pathogens. Measurements resemble fingerprints made up of the masses of the proteins present in the bacteria, mainly ribosomal proteins. These are usually highly conserved between species [28,29,30]. The aim of this analysis was to detect differences in the mass peaks and therefore in the protein profile of both strains. Although some differences could be seen, these were not significant (*p* < 0.05) in the statistical comparison. Therefore, if a hypothetical adaptation would have taken place, this would not have caused main changes involving the proteins displayed on the MALDI-TOF MS profile. The results of the genotypic analysis confirmed no differences in the ribosomal proteins.

PFGE *Xba*I patterns from both *S.* Choleraesuis strains have a similarity of 71.4%. This value is low [31] if taking into account that both isolates compared belong to the same serovar. Both band patterns differ strongly by showing a disparity in a total of eight bands [32,33]. Differences in the “H”-Antigen Phase between both strains might also have influenced this result. The strain of plant origin was phenotypically monophasic (6,7:-:1,5), lacking Phase 1 of the “H”-Antigen (c). Nevertheless, Next Generation Sequencing (NGS) of this strain showed genes encoding both of the “H”-Antigens were present in the genome [17]. Monophasic *S*. Choleraesuis strains, lacking the phase 1 “H” antigen c, are commonly associated with the *S.* Choleraesuis variant Kunzendorf and are frequently isolated from wild boars in Germany (oral communication, BfR). Furthermore, a broader comparison with other *S.* Choleraesuis var. Kunzendorf strains collected at the German National Reference Laboratory for *Salmonella* (data not shown) revealed that the animal strain with serotyp 6,7:c:1,5 showed less similarity in its PFGE band pattern to this collection than the plant strain, which showed a high similarity to the patterns from other *S.* Choleraesuis var. Kunzendorf isolates (BfR, personal communication). Our PFGE results confirmed that PFGE was discriminative for differentiation between different *S*. Choleraesuis strains but could not be accounted for evidencing any adaptions of the plant-internalized strain.

SNPs are valuable molecular markers for learning about the evolutionary relationship between strains [34,35]. The resulting distance of 2081 SNPs between both strains underlines that both strains are not closely related, despite belonging both to the serovar *S*. Choleraesuis. Likewise, previous studies comparing different strains *S.* Typhimurium could differ in exceptional cases even in more than 12,000 SNPs [36].

As the pangenome analysis showed, the strains compared in this study belong to the same serovar and variant, and share a high amount of core genes. Additionally, many genes were identified by means of the presence/absence analysis as alleles of the same gene and both strains shared at least one of these alleles. No differences in the main ribosomal proteins were seen, which would again confirm the results of the analysis by means of MALDI-TOF MS.

A total of 16 genes were present in only one of the strains. For example, the subset of genes *bcsA, bcsB, bcsE* and *bcsQ*, which are involved in the cellulose biosynthesis, were only present in the strain of animal origin. Gene *bcsA* is the first one in the operon and is normally highly conserved, while the second gene *bcsB* is less conserved [37]. The gene *bcsE* was shown to encode a c-di-GMP receptor required for optimal cellulose biosynthesis [38] and *bcsQ* encodes a putative MinD/ParA-like ATPase whose role in cellulose production remains unknown, although it might involve proper positioning of the enzyme complex [39]. Bacterial cellulose is required in many bacteria for biofilm formation, stress protection or an anti-virulence phenotype [40,41,42,43]. Studies with *S*. Enteritidis cellulose-deficient mutants suggested that cellulose production and biofilm formation would be important for survival in this species on surface environments [44]. Notabley, biosynthesis of cellulose is carried out by at least three different operon classes [45] and both analyzed *S.* Choleraesuis strains shared the genes *bcsC*, *bcsG* and *bcsZ*, the latter being responsible for the repression of cellulose production, which showed to enable efficient colonization of *S.* Typhimurium [43]. Although functions of some accessory genes of cellulose biosynthesis are still poorly understood [46] and the influence of the absence of these genes on the plant-internalized strains is not clear, it might be possible that biofilm formation is not necessary for internalization or survival in the plants.

In addition, the gene *smfF* encoding a putative fimbrial-like protein was only present in the plant-internalized strain. Fimbriae are one of the many known potential pathogenicity determinants shared among Enterobacteria. They are implicated in motility, aggregation and attachment to host cells. Especially genes associated with motility and adhesion have already been related to colonization and survival in or around plants [47]. For instance, the German *Escherichia coli* O104:H4 outbreak strain of 2011 was shown to have acquired an extra plasmid containing the aggregative adherence fimbriae type I (AAF/I) locus [22]. More than 10 fimbrial operons have been identified in *Salmonella* genomes with variations between serotypes. Different combinations of adhesins influence the ability to adhere to different cell types, which would then enable the colonization of new niches or hosts. However, the absence of only one fimbriae type should normally not significantly reduce the virulence of the affected *Salmonella* strain [48] and might therefore not pose an exceptional adaptation to the plant environment.

A previous study could identify, by means of Transposon insertion sequencing, that persistence of *Salmonella* Typhimurium on tomatoes required a different set of metabolic functions than during animal colonization or phytopathogens [49]. Additionally, further investigation allowed the identification of a new gene, *papA*, in a *S.* Newport strain isolated from tomatoes, which was responsible for fitness in this product [50]. Therefore, further characterization of this strain by means of this method might provide a better insight into these processes.

Although both sequenced genomes could not be closed, with a size of 4,723,419 bp for the plant-internalized strain and 4,733,941 bp for the animal strain, both genomes are close to the size of a complete genome and the difference in the number of base-pairs is minimal. Therefore, the probability of being one of these “missed” genes present in the small genetic gap would remain minimal.

## 5. Conclusions

Overall, the phenotypical analysis showed no main differences between a strain isolated from plant tissue and a randomly chosen strain from animal origin. This might suggest that any *Salmonella* strain could be able to internalize in plants or their fruits, like the plant-internalized strain analyzed in this study. Although some genetic differences were found, it remains unclear whether these were part of an evolutionary adaptation to their environments or part of a random casual event. Further analysis by means of transposon sequencing could provide a better insight into these genetic processes.

In case these results were conclusive and no adaptation would be needed for colonization of plants by human or animal *Salmonella* strains, direct superficial contamination of fresh produce after contamination via water, manure or soil would pose an important risk for food safety. This contamination might simply superficially affect plants. However, this could also result in the internalization of the microorganisms without an adaptation or mutation step being necessary. Thus, these bacteria would internally persist in the food items and could not be removed by further hygienic measures. By raw consumption of these products, internalized microorganisms would directly be ingested. Surface contaminating microorganisms are able to multiply under certain conditions on fresh produce and although colony counts of internalized bacteria were reported to be generally low, processing steps, such as cutting into pieces and subsequent storage under incorrect temperatures, might lead to a rapid multiplication of internalized bacteria. Altogether, this could mean an important increase in the risk of infection for consumers.

## Figures and Tables

**Table 1:** Genes identified only in one of the analyzed *Salmonella* Choleraesuis strains by means of Roary and their function.

Gene	Coding Function	*S.* Choleraesuis Plant Origin	*S.* Choleraesuis Animal Origin
*bcsA*	Cellulose synthase catalytic subunit [UDP-forming]		x *
*bcsB*	Cyclic di-GMP-binding protein		x
*bcsE*	Cyclic di-GMP-binding protein		x
*bcsQ*	Cellulose biosynthesis protein		x
*chiQ*	Putative lipoprotein		x
*csbX*	Alpha ketoglutarate permease	x	
*ifcA*	Fumarate reductase flavoprotein subunit		x
*lpfC*	Putative outer membrane usher protein	x	
*malS*	Periplasmic alpha-amylase	x	
*mppA*	Periplasmic murein peptide-binding protein	x	
*narW*	Putative nitrate reductase molybdenumcofactor assembly chaperone		x
*patB*	Cystathionine beta-lyase		x
*sfmF*	putative fimbrial-like protein	x	
*yfdC*	Inner membrane protein	x	
*yhjR*	Protein		x
*ylcG*	Putative protein	x	

* Gene presence confirmed for the correspondent strain by means of Roary.
